# Editorial: Machine learning, epistasis, and protein engineering: From sequence-structure-function relationships to regulation of metabolic pathways

**DOI:** 10.3389/fmolb.2022.1098289

**Published:** 2022-12-02

**Authors:** Frederic Cadet, Emma Saavedra, Per-Olof Syren, Brigitte Gontero

**Affiliations:** ^1^ Laboratory of Excellence LABEX GR, DSIMB, Inserm UMR S1134, University of Paris City and University of Reunion, Paris, France; ^2^ PEACCEL, Artificial Intelligence Department, Paris, France; ^3^ Department of Biochemistry, Instituto Nacional de Cardiología Ignacio Chávez, Mexico City, Mexico; ^4^ Science for Life Laboratory, School of Engineering Sciences in Chemistry, Biotechnology, and Health, KTH Royal Institute of Technology, Stockholm, Sweden; ^5^ Department of Fibre and Polymer Technology, School of Engineering Sciences in Chemistry, Biotechnology and Health, KTH Royal Institute of Technology, Stockholm, Sweden; ^6^ Aix Marseille University, CNRS, UMR7281 Bioénergétique et Ingénierie des Protéines, Marseille, France

**Keywords:** epistasis, non-linear interactions, machine learning, artificial intelligence, RNA enzyme, Crohn ‘s disease, antibiotic resistance, Protein-protein interaction networks

Epistasis is a term originating from genomics and describes the non-additivity of effects of gene interactions on functional parameters (Phillips, 2008). Both within and between genes, epistasis plays a fundamental role in the ability of protein sequences to evolve (Breen et al., 2012).

In protein sciences, epistasis reflects non-linearity effects, ie., non-additive impacts, resulting from interactions between mutations within a protein sequence (Reetz, 2013). The evolution of proteins cannot be understood without the knowledge of the epistasis phenomena (non-additive mutational effects) that take place within them. Indeed, epistasis can reverse the effect of a mutation from beneficial to deleterious. Likewise, thanks to epistasis phenomena, the conservation of a neutral mutation during evolution can lead to beneficial effects of greater amplitude. Interactions between mutated amino acids, as well as intramolecular interaction networks that can be set up following mutations, condition the function (Acevedo-Rocha et al., 2021). While it is clear that understanding sequence-structure-function relationships, and in particular intramolecular epistasis, is of paramount importance in protein engineering, the means to predict these phenomena are currently limited. Non-linear interactions are still poorly understood, making the design of networks of interacting amino acid residues serving to introduce desired functionality in an enzyme a bottleneck.

The prediction of protein structure has been an on-going challenge for computational methods, including artificial intelligence. However, the recent development of structure prediction deep learning (DL) tools such as alphafold2 (Jumper et al., 2021), ESMfold (Lin et al., 2022) or ProteinMPNN (Dauparas et al., 2022), has the potential to revolutionize this area (de Brevern, 2022; Goulet and Cambillau, 2022). Nevertheless, these DL tools are not suitable for predicting how individual amino acid changes alter protein structure and function (Eisenstein, 2021): they can’t predict epistatic effects.

After protein folding powered by DeepMind, Meta and/or Baker’s team, the next challenge is to accurately predict epistasis ie., the impact of non-linear interactions of mutations within the protein sequence. A recent review of machine learning and deep learning strategies examines how epistatic effects influence the success rate of protein engineering projects by comparing fifteen state-of-the-art approaches and provides a general workflow for non-experts when using such learning strategies (Cadet et al., 2022).

The aim of this Research Topic is to give an overview of recent advances and to discuss the current understanding of epistatic phenomena. Particular attention is paid to better understanding and modeling of epistatic phenomena that can impair the prediction of a property of interest.

The articles in this Research Topic illustrate the role of non-linear interactions between players through four particularly interesting examples: negative epistasis for an RNA enzyme; higher-order interactions in complex phenotypes revealed by the BowSaw tool; synergistic effect between Lysine acetylation levels, mSWI/SNF activity and BRD9 inhibition in many cellular contexts, and positive epistasis during the acquisition of resistance of pathogenic bacteria to antibiotics.

## Each of these articles is summarized below

Machine learning approaches based on large experimental datasets allow the behavior of some complex biological systems to be predicted. As we know, the rational design of RNA enzyme (ribozyme) activity is challenging, and many ribozyme-based systems are only engineered or improved by random mutagenesis and selection (*in vitro* evolution). Moreover, extensive pairwise and higher-order epistasis prevent straightforward prediction of the effect of multiple mutations that is needed for rational design. Beck et al. used high-throughput experimental data from variants of a self-cleaving ribozyme to train a predictive model through machine learning approaches. Using only sequence and activity data, they showed that a machine learning approach can be used for RNA design even for RNA molecules with unknown structures. This work is very important because self-cleaving ribozymes can be used to engineer control of gene expression thanks to their capacity to alter RNA processing and stability. Beck et al. unveiled negative epistasis in the RNA data suggesting that additional information, eg. thermodynamic stability of helices, might be necessary for increasing accuracy when predicting effects of long-range distance mutations. This will enable a more comprehensive understanding of RNA fitness landscapes for studying evolution and for guiding RNA-based engineering efforts.

Machine learning also enables the inference and classification of cellular, organismal and ecological phenotypes based on large datasets, e.g., from genomic, transcriptomic and metagenomic analyses. DiMucci et al. have developed a suite of algorithms, named BowSaw, that takes into account different combinations of variables also called “rules”. They applied this approach to study the role of the gut microbiome in Crohn’s disease. They found a previously unreported combination of microbial taxa that is broadly and precisely associated with Crohn’s disease samples. This is an important work whose current implementation shows that BowSaw can be applied to other datasets and used to uncover patterns associated with other diseases. This new suit of algorithms utilizes variable interactions in a trained Random Forest (RF) model in order to extract multiple candidate explanatory rules. Applying BowSaw to a study on the role of the gut microbiome in Crohn’s disease, DiMucci et al. shows that it can find a previously unreported combination of microbial taxa that is broadly and precisely associated with Crohn’s disease samples in the data set: they unveil higher-order interactions in complex phenotypes.

Protein-protein interaction networks are often affected by post-translational modifications. Since proteins rarely act alone, these interactions and their regulation play key roles in a cell. For example, acetylated histones not only affect chromatin condensation but also act as anchor points for bromodomain (BRD)-containing adapter proteins. These domains, and the proteins containing them, can act as chromatin scaffolds that organize large interaction networks which regulate transcription. Loehr et al. have created a cellular model allowing the study of lysine acetylation-dependent protein interaction networks. Using cell lines in which histone acetylation was dependent on acetate supplementation, they showed that the loss of lysine acetylation remodels the composition of the chromatin and the protein-protein interactions of BRDs. Their model may help to understand the functional characterization of BRD-containing proteins across distinct cellular contexts. Synergy between Kac levels, mSWI/SNF activity and BRD9 inhibition appears to be present in many cellular contexts. Loehr et al. created an inexpensive and flexible cellular model allowing the study of Kac-dependent protein interaction networks.

The increasing resistance of pathogenic bacteria to antibiotics is a major challenge to the health of humans, livestock and wildlife. One approach to overcome resistance is to use drug combinations. However, Mehta et al. showed that in the case of the pathogen *Francisella tularensis*, exposure to two different drugs, sequentially or in combination, resulted in a generalist mutation followed by further mutations that alternated between adaptation to one drug or the other, hence overcoming resistance. Mehta et al. showed that *c*lonal interference, weak pleiotropy and positive epistasis also contributed to combinatorial evolution. The finding suggests that the use of this non-interacting drug pair against *F. tularensis* may render both drugs ineffective because of mutational switch-backs that accelerate evolution of dual resistance. A better understanding of how drug combinations affect adaptation to multi-drug resistance is required to help overcome antibiotic resistance.

Our understanding of how to account for the effects of genetic variation and in particular the impact of non-linear interactions both within and between genes, is still in its infancy (Cadet et al., 2022; Wittmund et al., 2022). As shown in the articles cited above, the fundamental question of epistasis must be approached in a transdisciplinary manner mixing theoretical approaches, data driven modeling and wet laboratory experiments.
